# Utilization of oral antidiabetic medications in Taiwan following strategies to promote access to medicines for chronic diseases in community pharmacies

**DOI:** 10.1186/s40545-015-0035-5

**Published:** 2015-04-25

**Authors:** Jason C Hsu, Dennis Ross-Degnan, Anita K Wagner, Ching-Lan Cheng, Yea-Huei Kao Yang, Fang Zhang, Christine Y Lu

**Affiliations:** School of Pharmacy and Institute of Clinical Pharmacy and Pharmaceutical Sciences, National Cheng Kung University, Tainan, Taiwan; Department of Population Medicine, Harvard Medical School and Harvard Pilgrim Health Care Institute, Boston, MA USA

**Keywords:** Interrupted time series, Oral antidiabetic medications, Chronic medication prescriptions, Taiwan

## Abstract

**Objectives:**

Taiwan’s National Health Insurance (NHI) has encouraged physicians to use “chronic medication prescriptions” for patients with stable chronic diseases since 1995. Patients are allowed to refill such prescriptions at community pharmacies for a maximum of three months’ supply of medications without revisiting the doctor. In 2006, NHI initiated strategies targeting the public, doctors, and healthcare facilities to enhance the overall rate of chronic medication prescriptions, aiming to achieve 30% by 2010. We examined prescribing and dispensing of oral antidiabetic drugs from 2001 to 2010, before and after the start of the promotion strategies for chronic medication prescriptions in 2006.

**Methods:**

Using outpatient care data from the NHI database and the interrupted time series design, we analyzed changes in rate of chronic medication prescriptions, share of prescriptions filled at community pharmacies, and share of reimbursed expenditures accounted by community pharmacies.

**Results:**

During 2001-2010, the rate of chronic medication prescriptions for diabetes increased steadily by about 3% per year (from 3.5% to 26.2%). Three years after the promotion strategies, there was a non-significant reduction of 8.7% (95% confidence interval [CI]: -17.35%, 0.05%) in the rate of chronic medication prescriptions but increases in prescription refills at community pharmacies and associated reimbursed expenditures: 12.8% (95% C.I.:1.66%, 23.98%) and 15.8% (95% C.I.: -1.35%, 33.02%) respectively.

**Conclusions:**

While rate of chronic medication prescriptions was not significantly affected by the 2006 promotion strategy, shares of prescriptions refilled at community pharmacies and associated expenditures increased slightly but significantly.

## Introduction

Diabetes mellitus has become a global epidemic [1-3] and is a major and growing cause of morbidity and mortality in most countries [4-7]. More than 346 million people worldwide (~7.7%) [8-10] and about 1.47 million people in Taiwan (~4.5%) [11] were estimated to have diabetes in 2010. The prevalence of type 2 diabetes has risen steeply. Main reasons for the increasing prevalence of type 2 diabetes (~90% of diabetes mellitus) include a more sedentary lifestyle, increased rates of obesity, improved diagnosis, longer life expectancy and aging populations [3,5,7,12].

Oral hypoglycemic medications are the mainstay of treating type 2 diabetes. New oral hypoglycemic medications -- thiazolidinediones and dipeptidyl peptidase inhibitors -- have become available over the last decade. Given the growing prevalence of type 2 diabetes, the availability of new but more expensive medications is likely to increase the overall costs of oral antidiabetic treatment [5] and the economic burden for public and private health care payers [6,13].

Understanding prescribing patterns can inform the development of interventions to promote appropriate use of medicines. Studies have reported the utilization of antidiabetic medications over time in the US [14,15], the UK [5,7,16], Italy [3], Germany [6,17], France [2], and Hungary [18]. In Taiwan, Chiang et al. [19] reported prescribing patterns of oral antidiabetic drugs from 1997 to 2003. However, no studies have reported recent trends in utilization of oral antidiabetic medications in Taiwan. Further, little is known about health policy impacts on access to oral antidiabetic agents.

Since 1995, Taiwan’s National Health Insurance (NHI) has encouraged physicians to use “chronic medication prescriptions” [20] for patients with stable chronic diseases. Special prescription forms can be used to indicate that prescribers allow prescriptions to be refilled once a month for two times for a total of three months’ supply of medications instead of a month’s supply only [21]. Using such prescriptions, patients can refill their prescription at insurance-contracted community pharmacies of their choice without revisiting a doctor’s office [21]. Community pharmacies contracted with NHI have the following key characteristics: (i) they are financially independent from any hospitals or clinics, but may collaborate with primary-care physicians; [22] and (ii) they can sell over-the-counter drugs, and dispense prescription drugs for patients presenting prescriptions from any hospitals or clinics (including chronic medication prescriptions) and be reimbursed by NHI (in contrast, hospital pharmacies can only dispense prescriptions from their related hospitals or clinics) [23]. The objectives of the promotion strategies for chronic medication prescriptions are to reduce unnecessary outpatient services and related expenses, including outpatient registration fees, medication copayments, and traveling expenses, and to enhance more convenient access to medicines for treatment of chronic disease for clinically stable patients.

In May 2006 Taiwan’s NHI initiated five-year strategies to promote the use of chronic medication prescriptions. The strategies involved efforts to: (1) continuously promote to patients the benefits of using chronic medication prescriptions through patient education; (2) regularly report rates of chronic medication prescriptions by healthcare institutions on the National Health Insurance Administration’s website for patients’ reference; (3) set the target rates of chronic medication prescriptions by end of 2006 (all healthcare institutions 15%; medical centers 24%; regional hospitals 14%; local hospitals 9%; and clinics 13%); and (4) aim to increase the overall rate of chronic medication prescriptions by 4% per year for all healthcare institutions and reach an average rate of 30% by 2010 [24].

The purpose of this study was to examine rates of chronic medication prescriptions and shares of prescription fills and reimbursed expenditures by hospitals vs. community pharmacies from 2001 to 2010, before and after the start of five-year promotion strategies in 2006. Our study focused on oral antidiabetic agents, which are important medications for diabetes, a major chronic illness in Taiwan.

## Methods

### Data sources

This study used claims data from Taiwan’s National Health Insurance Research Database (NHIRD). The database includes information from a nationwide, mandatory-enrollment and single-payer healthcare system created in 1995. Nearly all of the Taiwanese population (around 23 million residents) is enrolled and this system contracts with 97% of hospitals and clinics throughout the country [25]. The NHI covers a wide range of prescription medicines, and inpatient and outpatient medical services [26]. We used claims data for outpatient care related to diabetes (International Classification of Diseases, 9^th^ edition, code 250.xx) from January 2001 to December 2010 extracted from NHIRD. For each prescription claimed, records actually indicate if the prescription used the “chronic medication prescription form”, representing that physicians allowed medications to be refilled at community pharmacies.

### Drugs of interest

Using the Anatomical Therapeutic Chemical classification system of the World Health Organization, we grouped oral hypoglycemic drugs into biguanides, sulfonylureas, alpha glucosidase inhibitors, thiazolidinediones, dipeptidyl peptidase 4 inhibitors, and others (e.g., guar gum, repaglinide, nateglinide). Our analysis focused on the first five classes because they accounted for 86.4%-99.2% of utilization and 93.3%-97.2% of expenditures.

### Outcome measures

To examine effects of the 2006 promotion strategies, we measured quarterly rates of chronic medication prescriptions for diabetes, defined as annual number of chronic medication prescriptions for diabetes divided by annual number of diabetic outpatient visits. This indicates uptake of chronic medication prescriptions by physicians (physician prescribing behavior). We also calculated the quarterly and yearly shares of all antidiabetic prescriptions refilled at community pharmacies. This is defined as aggregated annual prescription volume in DDDs for diabetes filled at community pharmacies divided by aggregated annual prescription volume in DDDs filled at both hospital and community pharmacies to indicate patients’ use of community pharmacies over time (patient dispensing/refilling behavior). Similarly we calculated shares of reimbursed expenditures for antidiabetic prescriptions accounted by community pharmacies (aggregated annual reimbursed expenditures accounted by community pharmacies divided by aggregated annual reimbursed expenditures accounted by both hospital and community pharmacies). We calculated the above measures for both overall and each class of antidiabetic agents.

To provide the context of utilization patterns of antidiabetic medications in Taiwan, we calculated quarterly and yearly prescription volume per patient and government-reimbursed costs per patient. Annual prescription volume per patient was defined as aggregated annual prescription volume in daily defined doses (DDDs) divided by annual number of diabetic patients. Similarly, annual government-reimbursed costs per patient was defined as aggregated annual expenditure of prescriptions for oral antidiabetic drugs divided by annual number of diabetic patients. We calculated these for each class of antidiabetic agents and for all oral antidiabetic drugs.

### Statistical analysis

We used the interrupted time series design [27], a strong quasi-experimental method, to examine impacts of the 2006 promotion strategies on rates of chronic medication prescriptions, proportion of prescriptions refilled at community pharmacies, and proportion of expenditures accounted by community pharmacies. We used segmented linear regression models to estimate post–policy changes in both the level and trend of these study outcomes [28-31]. Using baseline trends, we projected rates over time with the assumption that the baseline trend reflected what would have happened without the implementation of the promotion strategies. The basic model included terms to estimate the baseline level for each outcome (intercept), baseline trend (slope), changes in the level immediately after policy implementation, and changes in trend after the policy [27,32]. Our models controlled for autocorrelation [33]. To identify the most parsimonious models, we used backward elimination and excluded non-significant terms (P > 0.05). To summarize results in a single metric, we estimated absolute and relative changes (with 95% confidence intervals, CI) [34] in outcomes at both 1 year and 3 years following the 2006 promotion strategies compared to projected rates. All analyses were carried out with SAS software, Version 9.3 (SAS Institute, Cary, NC).

## Results

### Utilization of oral antidiabetic medications over time

During the 10-year study period, the estimated number of patients with type 2 diabetes in Taiwan increased from 527,045 in 2001 to 1,025,646 in 2010, an average rate of 10.5% increase per year, while the Taiwanese population increased from 22.4 million in 2001 to 23.0 million in 2010, an average annual rate of 0.3% [35].

The volume of oral antidiabetic drugs prescribed increased from 585.9 DDDs per patient per year in 2001 to 650.5 DDDs per patient per year in 2004, but decreased slightly after 2007 to 635.6 DDDs per patient per year in 2010. Similarly, expenditures on oral anti-diabetic drugs increased from US$162.8 per patient per year in 2001 to US$241.64 per patient per year in 2004, then slightly decreased after 2006 to US$195.34 per patient per year in 2010 (see Table 1).

**Table 1:** **Population trends in use of oral antidiabetic drugs**

	**2001**	**2002**	**2003**	**2004**	**2005**	**2006**	**2007**	**2008**	**2009**	**2010**
**Overall**										
Patient Number	527,045	579,017	624,540	689,423	736,743	784,132	843,334	903,947	969,161	1,025,646
Volume/patient (DDDs)_c._	585.94	613.85	640.25	650.54	652.36	651.68	653.71	637.1	630.16	635.59
Expenditure/patient (US$)_d._	162.80	199.99	219.48	241.64	233.55	232.84	216.31	207.33	205.63	195.34
**Subgroups by class**										
**Volume/patient (DDDs)**_c._										
Biguanides	125.11	127.77	129.51	133.68	137.99	141.71	144.18	144.81	143.07	138.19
Sulfonylureas	449.33	453.46	458.54	444.33	421.16	410.44	399.74	379.16	354.72	325.44
Alpha-glucosidase inhibitors	3.64	5.97	10.72	13.81	14.67	16.13	18.04	19.35	19.34	19.21
Thiazolidinediones	3.23	16.04	27.48	34.15	31.15	32.64	36.64	37.44	36.66	38.4
Dipeptidyl peptidase-4 inhibitors	N/A _b._	N/A	N/A	N/A	N/A	N/A	N/A	N/A	10.17	27.40
Others_a._	4.63	10.61	14.00	24.56	47.38	51.12	55.12	56.35	66.21	85.93
**Expenditure/patient (US$)**_d._										
Biguanides	51.20	51.67	46.46	46.73	46.99	44.42	34.65	32.88	32.47	28.97
Sulfonylureas	94.38	98.10	95.10	98.36	96.40	94.07	85.00	81.35	74.84	59.13
Alpha-glucosidase inhibitors	4.94	7.41	12.46	15.81	16.52	17.87	18.54	18.45	17.39	15.06
Thiazolidinediones	7.70	33.85	53.87	66.64	60.65	62.64	64.40	60.83	55.67	48.42
Dipeptidyl peptidase-4 inhibitors	N/A	N/A	N/A	N/A	N/A	N/A	N/A	N/A	11.68	31.13
Others_a._	4.59	8.97	11.59	14.10	12.99	13.84	13.72	13.80	13.58	12.63

a. Others: guar gum, repaglinide, nateglinide, exenatide, liraglutide, mitiglinide.

b. N/A = not applicable.

c. Volume/patients (DDDs) = aggregated annual volume (DDDs) of prescription/annual number of diabetic patient.

d. Expenditure/patient (US$) = aggregated annual expenditure (US$) of prescription/annual number of diabetic patient.

**Figure 1:**
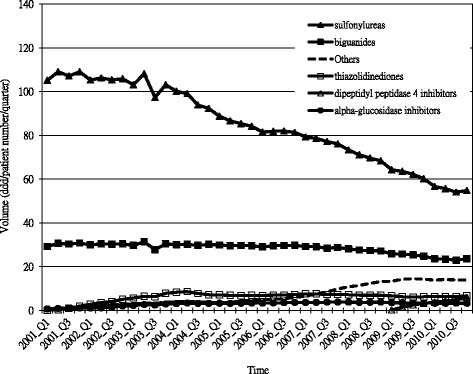
Volume of oral antidiabetic drug prescriptions filled at hospitals and community pharmacies over time.

By drug class, sulfonylureas and biguanides, which are generally recommended as first-line therapies, dominated the Taiwan market for diabetic management over the last decade. The use of biguanides remained steady (averaging 136.6 DDDs per patient per year during the period 2003 to 2010). However, sulfonylureas gradually decreased from 458.5 DDDs per patient per year in 2003 to 325.4 DDDs per patient per year in 2010, while use of newer classes of antidiabetic medications (alpha-glucosidase inhibitors, thiazolidinediones, dipeptidyl peptidase 4 inhibitors) increased. Figure 1 shows the volumes of oral antidiabetic drugs refilled by hospital and community pharmacies over time separately. The volume refilled by community pharmacies increased steadily for each class of medications during this period.

### Effects of strategies on rates of chronic medication prescriptions

Table 2 presents the rate of chronic medication prescriptions, share of prescriptions refilled at community pharmacies, and share of reimbursed expenditures accounted by community pharmacies annually over time. During 2001-2010, the overall rate of chronic medication prescriptions (3.54%-26.24%), the share of prescriptions refilled at community pharmacies (3.67%-31.30%), and the share of reimbursed diabetes medication expenditures accounted by community pharmacies (3.36%-32.35%) increased steadily at an average rate of around 3% per year. Classes of medications showed similar growth rates for share of prescriptions refilled at community pharmacies and share of reimbursed expenditures accounted by community pharmacies during the study period. Examining share of prescriptions refilled at community pharmacies and share of reimbursed expenditures accounted by community pharmacies revealed similar findings.

**Table 2:** **Rates of chronic medication prescriptions**_**a.**_ **and prescriptions filled at community pharmacies over time**

		**2001**	**2002**	**2003**	**2004**	**2005**	**2006**	**2007**	**2008**	**2009**	**2010**
**Overall**											
	Rate of chronic medication prescriptions for diabetes (%)_d._	3.54%	5.65%	9.40%	12.84%	15.77%	19.94%	23.13%	25.29%	26.55%	26.24%
	Share of prescriptions filled at community pharmacies (%)_e._	3.67%	5.52%	8.38%	10.40%	14.20%	16.68%	19.37%	23.46%	28.14%	31.30%
	Share of reimbursed expenditures accounted by community pharmacies (%)_f._	3.36%	4.97%	7.11%	9.30%	13.71%	16.23%	19.51%	24.48%	29.22%	32.35%
**Subgroups by class**											
	**Share of prescriptions filled at community pharmacies (%)**_e._										
	Biguanides	3.43%	5.31%	8.39%	10.44%	14.12%	16.73%	19.52%	23.32%	28.22%	31.66%
	Sulfonylureas	4.01%	6.22%	9.59%	11.80%	15.58%	17.96%	20.17%	24.06%	28.42%	31.29%
	Alpha-glucosidase inhibitors	0.94%	1.98%	3.17%	6.14%	10.27%	13.38%	17.06%	22.02%	27.48%	30.95%
	Thiazolidinediones	1.21%	2.93%	4.66%	6.81%	11.23%	13.67%	18.18%	23.91%	29.23%	32.00%
	Dipeptidyl peptidase-4 inhibitors	N/A _c._	N/A	N/A	N/A	N/A	N/A	N/A	N/A	24.89%	30.32%
	Others_b._	1.30%	1.51%	3.40%	5.30%	8.87%	11.99%	15.25%	20.12%	25.42%	28.59%
	**Share of reimbursed expenditures accounted by community pharmacies (%)**_f._										
	Biguanides	3.23%	5.07%	7.71%	9.98%	13.91%	16.92%	20.47%	24.77%	29.51%	32.91%
	Sulfonylureas	3.85%	6.24%	9.49%	12.06%	16.61%	18.85%	21.13%	25.26%	29.73%	32.80%
	Alpha-glucosidase inhibitors	0.75%	1.67%	2.78%	5.46%	9.47%	12.72%	16.47%	21.77%	27.63%	31.36%
	Thiazolidinediones	1.12%	2.93%	4.29%	6.56%	11.18%	13.76%	18.62%	24.88%	30.28%	33.23%
	Dipeptidyl peptidase-4 inhibitors	N/A	N/A	N/A	N/A	N/A	N/A	N/A	N/A	25.81%	31.13%
	Others_b._	1.28%	1.38%	2.99%	5.11%	8.71%	11.88%	15.38%	20.97%	26.41%	29.80%

a. Chronic medication prescriptions allow prescriptions to be refilled once a month for two times for a total of three months’ supply of medications, without repeat visits to a doctor’s office.

b. Others: guar gum, repaglinide, nateglinide, exenatide, liraglutide, mitiglinide.

c. N/A = not applicable.

d. Rate of chronic medication prescriptions for diabetes = annual number of chronic medication prescriptions for diabetes/annual number of diabetic outpatient visits.

e. Share of prescriptions filled at community pharmacies = aggregated annual volume (DDDs) of prescription filled at community pharmacies/aggregated annual volume (DDDs) of prescription filled at both hospital and community pharmacies.

f. Share of reimbursed expenditures accounted by community pharmacies = aggregated annual reimbursed expenditures accounted by community pharmacies/aggregated annual reimbursed expenditures accounted by both hospital and community pharmacies.

**Figure 2:**
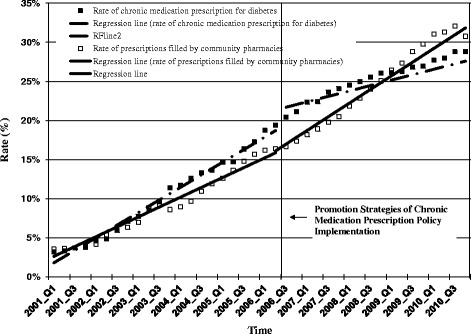
Quarterly proportions of chronic medication prescriptions for diabetes and of prescriptions refilled at community pharmacies, 2001-2010.

Table 3 details parameter estimates from segmented regression models of changes in outcomes following the promotion strategies. Figure 2 shows the changes in rate of chronic medication prescriptions and the share of prescriptions refilled at community pharmacies over time.

**Table 3:** **Estimated changes (with 95% confidence interval**_**a.**_ **) in use of oral antidiabetic products following the 2006 five-year chronic medication prescriptions promotion strategy**

	**Intercept**	**Baseline trend**	**Level change**	**Trend change**	**Absolute change (1 year later)**	**Relative change (1 year later)**	**Absolute change (3 years later)**	**Relative change (3 years later)**
**Rate of chronic medication prescriptions for diabetes**_b._	0.0100	0.0080 (0.00069, 0.0091)	0.0312 (0.0119, 0.0505)	-0.0046 (-0.0063, -0.0029)	1.28% (-0.68%, 3.24%)	6.08% (-3.62%, 15.78%)	-2.38% (-4.98%, 0.22%)	-8.65% (-17.35%, 0.05%)
**Share of prescriptions filled at community pharmacies**_c._	0.0200	0.0063 (0.0053, 0.0073)		0.0024 (0.0005, 0.0043)	0.97% (0.22%, 1.73%)	5.49% (0.79%, 10.19%)	2.92% (0.66%, 5.19%)	12.82% (1.66%, 23.98%)
**Share of reimbursed expenditures accounted by community pharmacies**_d._	0.0173	0.0063 (0.0047, 0.0079)		0.002965 (0.0002, 0.0057)	1.19% (0.08%, 2.30%)	6.80% (-0.45%, 14.05%)	3.56% (0.23%, 6.89%)	15.83% (-1.35%, 33.02%)

a. 95% CI = estimate +/- (1.96*se); All terms p < 0.05 retained in models.

b. Rate of chronic medication prescriptions for diabetes = annual number of chronic medication prescriptions for diabetes/annual number of diabetic outpatient visits.

c. Share of prescriptions filled at community pharmacies = aggregated annual volume (DDDs) of prescription filled at community pharmacies/aggregated annual volume (DDDs) of prescription filled at both hospital and community pharmacies.

d. Share of reimbursed expenditures accounted by community pharmacies = aggregated annual reimbursed expenditures accounted by community pharmacies/aggregated annual reimbursed expenditures accounted by both hospital and community pharmacies.

The rate of chronic medication prescriptions gradually rose from 3% in the first quarter of 2001 to 19% in the second quarter of 2006. After the promotion strategies were implemented in the third quarter of 2006, there was a small increase in level (3.12%, 95% CI: 1.19% ~ 5.05%) but a small reduction in trend (-0.46% per quarter, 95% CI: -0.63% ~ 0.29%). Compared to expected rates, these represented an increase of 6.08% (95% CI: -3.62% ~ 15.78%) at 1 year post-policy but a reduction of 8.65% (95% CI: -17.35% ~ 0.05%) at 3 years post-policy.

The rate of prescriptions filled at community pharmacies gradually grew from 4% in the first quarter of 2001 to 16% in the second quarter of 2006. After the promotion strategies, there was a small increase in trend (0.24% per quarter, 95% CI: 0.05% ~ 0.43%), leading to statistically significant increases of 5.49% (95% CI: 0.79% ~ 10.19%) at 1 year post-policy and 12.82% (95% CI: 1.66% ~ 23.98%) at 3 years post-policy compared to expected rates.

Following the promotion strategies, reimbursed expenditures accounted by community pharmacies were 6.80% (95% CI: -0.45% ~ 14.05%) higher at 1 year post-policy compared to expected rates, and 15.83% (95% CI: -1.35% ~ 33.02%) higher than expected at 3 years post-policy.

## Discussion

This study examined the long-term effects of a policy change in Taiwan to reduce costs and enhance management of chronic diseases – the 2006 strategies to promote chronic medication prescriptions – on physician’s prescribing behavior (rate of chronic medication prescriptions) and patient’s prescription refill behavior (share of prescription refills at hospital pharmacies vs. community pharmacies) for diabetes.

Between 2001 and 2010, the number of physician encounters with patients treated for diabetes in Taiwan increased almost two-fold, possibly reflecting better access to care and better diagnosis of diabetes over time. The annual volumes and expenditures of antidiabetic medications per capita were relatively stable during this period. Our findings of a decline in volume (DDDs) of sulfonylureas and an increase in volume of biguanides in Taiwan are consistent with the majority of existing articles in several countries over similar time periods, including the US [14], UK [16], France [2], Germany [6,17] and Italy [3]. The launch of new medications, alpha-glucosidase inhibitors and thiazolidinediones, provided physicians and patients more choices for diabetes management. Trends in use of oral antidiabetic medications from 2001 to 2010 may be influenced by several interventions and events. Examples include the launch of new medications, new guidelines, patent expires, introduction of generic products, and changes in reimbursement-related policies [30].

Our findings indicate that the rate of chronic medication prescriptions for diabetes rose steadily between 2001 and 2010. Since 1995, the Taiwan’s National Health Insurance Administration has allowed use of chronic medication prescription forms by doctors for patients whose treatments for their chronic condition have stabilized. Theoretically, increases in uptake of chronic medication prescriptions should benefit both patients and physicians. Patients save time spent traveling to visit physicians and waiting time in offices, and also reduce their financial burdens for fees associated with visits to doctors’ offices [36]. Physicians can spend more time with patients whose condition is less stable, and thereby possibly improve quality of care. For the healthcare system, the promotion strategies intended to reduce use of and spending on non-essential medical services. While chronic medication prescriptions may reduce physician visits and thus physician-patient encounters and communications, instead it may enhance communication between pharmacists and patients. Previous studies found a positive association between chronic medication prescriptions and medication access and adherence, and thereby a better control of chronic diseases [37,38].

Our longitudinal study showed that the rate of chronic medication prescriptions for diabetes did not increase appreciably following the 2006 promotion strategies. In fact, our estimate at 3 years post-policy was a reduction of 8.65%, and overall the rate fell slightly short of the target rate of 30%. This finding may not be surprising because the goal of chronic medication prescriptions was to enhance the convenience of refilling prescriptions by patients with stable disease [37], and the growth in chronic medication prescriptions was likely to slow down at some point after reaching the majority of patients with stable diabetes. Another possible explanation might be that the rate of chronic medication prescriptions has already reached the annual target rates; thus there was no need to prescribe using chronic medication prescription forms. Moreover, chronic medication prescriptions may reduce physician incomes due to foregone visit fees.

Our measures of proportion of prescription refilled at community pharmacies and associated expenditures indicate patient refilling behavior. In contrast to rates of chronic medication prescriptions, the share of prescriptions refilled at community pharmacies not only grew between 2001 and 2006, it increased at a higher rate after the promotion strategies. Patients appear to be increasingly motivated to refill prescriptions at community pharmacies. Possible benefits to patients include convenience, reduced cost, and more time to consult pharmacists when they fill a prescription at community pharmacies, which might result in increased accuracy in taking medicine and decreased occurrence of drug-induced side effects [39,40]. Given their increasing importance in filling prescriptions, community pharmacists also have the potential to become more actively involved in evaluating and tracking health outcomes. These potential outcomes should be examined in future studies.

The continually increasing share of reimbursed expenditures accounted by community pharmacies may have economic implications beyond what we examined. For example, community pharmacies have likely gained increased profits by dispensing more prescription medications. In contrast, the policy may have impacted hospital pharmacies’ operating income and management strategies due to a reduced number of prescription refills.

Many countries allow multiple times of prescription refills at community pharmacies for patients with chronic illnesses to enhance medication access and adherence, for example, the US and Australia. However, despite allowing multiple months of supply of medications, patient cost-sharing has been a major factor associated with poor medication access and adherence in the US [41]. Apart from economic pressures, medication related beliefs (e.g., perceived necessity) also influence patients’ medication use behavior [42]. In Australia where patient cost-sharing is less of a concern, factors associated with medication non-adherence include perceived treatment inefficacy and unfavorable side effects [43]. Experiences in other countries suggest that other interventions may need to be combined with chronic medication prescriptions to enhance medication access and adherence in Taiwan.

This study has several limitations. Our interrupted time series design is robust to most threats to internal validity; a possible limitation is a co-intervention (an intervention that occurred at the same time as the 2006 strategies and is related to the outcomes measured). However, our review of policy documents showed no interventions occurred at the same time as the promotion strategies [44]. Second, our measures using insurance claims data do not necessarily reflect actual drug consumption by patients, similar to other studies using such data sources. Third, we examined only oral antidiabetic medications. Further studies are needed to understand the effects of the 2006 promotion strategies on use of medications for other chronic conditions such as hypertension as well as medication adherence and patient satisfaction with care. Fourth, we did not separately analyze incident versus prevalent diabetes patients. The case-mix of such populations may explain the small difference in share of prescriptions filled at community pharmacies between the different drug classes. Finally, we estimated rates of chronic medication prescriptions using insurance claims data rather than using electronic medical records data, thus may have underestimated the true rates (sensitivity); however, our interrupted time series design is able to detect changes in rate, adjusting for previous trends; therefore, the change estimated would be in the right direction. Notwithstanding these limitations, this study provided valuable insights into changes in physicians’ prescribing behavior (uptake of chronic medication prescriptions) and patients’ prescription refill behavior (refills at hospitals vs. community pharmacies) for diabetes care following the 2006 strategies to promote chronic medication prescriptions in Taiwan.

## Conclusion

With its aging population, rapid growth in the prevalence of chronic diseases is a challenge in Taiwan. Continued, affordable, equitable access to medications for patients with stable chronic diseases plays an important role in quality of care and prevention of complications from diabetes. This study examined effects of NHI strategies, implemented in 2006 for five years, to enhance access to medications for chronic diseases. We focused on medications for diabetes, a major chronic condition in Taiwan. While rate of chronic medication prescriptions was not significantly affected by the 2006 promotion strategies, shares of prescriptions refilled at community pharmacies and reimbursed expenditures accounted by community pharmacies increased slightly but significantly.
